# Mood Responses and Regulation Strategies Used During COVID-19 Among Boxers and Coaches

**DOI:** 10.3389/fpsyg.2021.624119

**Published:** 2021-03-05

**Authors:** Reece J. Roberts, Andrew M. Lane

**Affiliations:** Research Centre for Sports Exercise Performance, University of Wolverhampton, Walsall, United Kingdom

**Keywords:** mental toughness, emotional intelligence, mood regulation, coach-athlete relationship, COVID-19

## Abstract

The COVID-19 pandemic brought unprecedented changes to daily life and in the first wave in the UK, it led to a societal shutdown including playing sport and concern was placed for the mental health of athletes. Identifying mood states experienced in lockdown and self-regulating strategies is useful for the development of interventions to help mood management. Whilst this can be done on a general level, examination of sport-specific effects and the experience of athletes and coaches can help develop interventions grounded in real world experiences. The present study investigated perceived differences in mood states of boxers before and during COVID-19 isolation in the first lockdown among boxers. Boxing is an individual and high-contact sport where training tends to form a key aspect of their identity. Boxers develop close relationships with their coach and boxing. Hence boxers were vulnerable to experiencing negative mood, and support via the coach was potentially unavailable. Participants were 58 experienced participants (44 boxers, male *n* = 33, female *n* = 11; 14 boxing coaches, male *n* = 11, female *n* = 3). Boxers completed the Brunel Mood Scale to assess mood before COVID-19 using a retrospective approach and during COVID-19 using a “right now” time frame. Boxers responded to open-ended questions to capture mood regulation strategies used. Coaches responded to open ended questions to capture how they helped regulate boxer’s mood. MANOVA results indicated a large significant increase in the intensity of unpleasant moods (anger, confusion, depression, fatigue, and tension) and reduction in vigor during COVID-19 (*d* = 0.93). Using Lane and Terry (2000) conceptual framework, results showed participants reporting depressed mood also reported an extremely negative mood profile as hypothesized. Qualitative data indicated that effective mood-regulation strategies used included maintaining close coach-athlete contact and helping create a sense of making progress in training. When seen collectively, findings illustrate that mood state responses to COVID-19 were severe. It is suggested that that active self-regulation and self-care should be a feature of training programmes to aid coaches and boxers in regulating mood when faced with severe situational changes.

## Introduction

The Coronavirus pandemic has resulted in over 2 million deaths worldwide by January 2021 (World Health Organisation, 2020). In the UK, from March 2020 restrictions on movements and social interaction, called a lockdown, were employed to reduce infection rates (Chatterjee and Chauhan, 2020), and as part of this strategy sporting events were canceled. In enforcing a lockdown, many experts using data from other pandemics predicted an increase in negative mental health (Brooks et al., 2020). Initial research in the UK indicated that an increase in negative mood as indicated by people reporting to feel stressed, scared and less happy once lockdown started (YouGov, 2020). Mood data collected online at the start of the COVID pandemic from 1,062 participants, where the location of participants was not identified, reflected an inverse iceberg profile. An iceberg profile is characterized by significantly elevated scores for tension, depression, anger, fatigue, and confusion, and below average scores for vigor; a profile associated with increased risk of mental health issues (Terry et al., 2020). Hence, there is growing evidence showing people experience negative mood during lockdown.

At the start of the lockdown in the UK (March 23rd, 2020), it was suggested that support staff should anticipate a greater demand for mental health provision for athletes during the ensuing months (Brooks et al., 2020; Chatterjee and Chauhan, 2020). A key issue is how this might be provided. On one hand, there is an argument for using common principles of self-care, and within the UK, one example of this was advising people to take 1 h of exercise daily amidst the general advice of staying at home (Brooks et al., 2020). Whilst such advice might help the general population, it is likely to be received differently by participants from specific sports. In sports such as boxing, which rely on equipment, coaches to organize and run sessions, this decision effectively closed formal training. The present study focuses on a single sport, namely boxing, a sport where there is a dearth research and where the effects of lockdown could be hard.

As COVID should associate with mood disturbance, available evidence suggests that boxers experience negative mood when training (Lane and Terry, 1996; Hall and Lane, 2001). Research has shown that mood states associated with performance in boxing (Lane and Lane, 2008) and that boxers experienced intense unpleasant mood (Hall and Lane, 2001). Professional boxing consultancy has reported using mood management to help identify positive adaptation to training in boxers (Lane, 2006). Whereby it was suggested that the coach was important in the management of mood among boxers (Lane, 2006).

Mood is defined as “a set of feelings, ephemeral in nature, varying in intensity and duration, and usually involving more than one emotion” (Lane and Terry, 2000). The Profile of Mood States model has been used for assessing mood, a tool that has been found to be useful for researchers and practitioners alike (McNair et al., 1971; Morgan, 1980; Lane and Terry, 2000). Mood profiling became popular following the claim that champion athletes tended to report an *iceberg* shaped profile (Morgan, 1980; Terry and Lane, 2000). However, a meta-analysis concluded that mood profiles may not predict champion athletes but can predict performance variations (Beedie et al., 2000). Mood profiling has utility beyond the realm of performance prediction. It can help monitor the effects of environmental changes (Lane et al., 2004). Mood profiling has been used to screen for potential clinical issues (Van Wijk et al., 2013) and monitoring the progress of cardiac rehabilitation patients in Brazil (Sties et al., 2014). The utility of mood to relate to performance and help detect poor mental health in a range of different areas of application as suggested above suggests it could be useful to assess in a context such as COVID-19, where athletes and coaches might have concerns over sport participation and performance, and where participation in sport is used to regulate their mood.

Recent research has identified new mood profiles and these have been also explored during COVID-19 (Terry et al., 2020). These are labeled the Inverse Everest, Shark Fin, Submerged, and Surface profiles (Parsons-Smith et al., 2017; Quartiroli et al., 2018; Han et al., 2020). The Inverse Everest profile is characterized by low vigor scores, high scores for tension and fatigue, and very high scores for depression, anger, and confusion. The Shark Fin profile is characterized by below average scores for tension, depression, anger, vigor, and confusion, combined with a high score for fatigue. The Submerged profile is characterized by below average scores for all six mood dimensions. The Surface profile is characterized by average scores for all six mood dimensions. It is believed that the identification of new profiles is helpful and will drive theoretical development on the interaction between mood states and help guide practitioners, however, the authors also suggest that some extra attention is given to the concept of depressed mood. The focus on depressed mood in a COVID-19 context seems particularly important given the dual effect of poor mental health and reduction in training and so potentially reduced fitness. Therefore, whilst such profiling techniques are useful, they might not consider or suitably emphasize certain mood states such as depression. The Inverse Everest profile is arguably the most concerning from a mental health perspective. Linked to this model is Lane and Terry’s conceptual mood which identifies depressed mood as an important mood state that influences the whole mood profile. Lane and Terry found that a small indicator of depressed mood associated with much larger scores in anger, confusion, fatigue and tension. This profile is different to the inverse iceberg model as depression scores are much lower. Arguably, identifying depressed mood at the earliest stage is desirable from an intervention perspective.

It should be noted that Lane and Terry referred to a depressed mood or emotional state rather than inferring a clinical condition. A key aspect of Lane and Terry’s theory is that a small indicator of depression, that is, a score of greater than zero using the Brunel Mood Scale (BRUMS) is all that is required (Lane and Terry, 2000). When seen collectively, there are good reasons to examine and assess mood states in a COVID-19 crisis and whilst it is expected that an inverse iceberg profile will be dominant, albeit one which is displayed with small amount of depression. A key aspect of research in the COVID-19 crisis should be on how people regulate their moods and how successfully they can do will influence their mental health.

In general life, in specific sports and in the COVID-19 pandemic, emotion regulation is an important concept. Emotion regulation is the automatic or deliberate use of strategies to initiate, maintain, modify or display emotions (Gross and Thompson, 2007; Ford and Gross, 2019). Emotion regulation is proposed to be part of a self-regulatory process in which individuals consciously or non-consciously monitor the intensity of emotions and develop strategies to increase, reduce or maintain the intensity of emotions to desirable levels (Lane et al., 2012).

In terms of how people regulate their feelings, studies indicate that people organically use conscious and non-conscious strategies to regulate their emotions with over 400 strategies identified (Lane and Terry, 2005). Examples of strategies include activities such as talking to people, watching television, going out for meals, playing sport, and listening to music. These are all tasks that people identify with as part of day-to-day living and unlikely to be consciously identified and used as mood regulation strategies until prompted to reflect on how they aid emotional well-being. In sport, research has identified that feelings are also regulated by athletes using psychological skills such as imagery, goal-setting and self-talk (Lane et al., 2012, 2016c). Psychological skills are typically used to manage performance, but where performance and emotional control are inter-related, they serve a dual goal. The close connection between strategies used to manage performance, such as how well training had gone and feeling physically prepared were evidenced in a recent study in running (Han et al., 2020). People are aware of their own mood states and can recognize the mood states of others and moreover, recognize that there are many benefits in having optimal mood states in goal striving settings (Lane et al., 2012).

When considering how athletes might regulate their mood states during training, it is important to identify what strategies are available due to lockdown restrictions. In sport, training will be affected and the extent to which it can help maintain mental health could worsen if the athlete cannot participate in their sport due to lack of availability of equipment or a place to train. A recent study offered general advice on the principles of conditioning for athletes and coaches to follow (Andreato et al., 2020). At a sport-specific level, in sports which require equipment, the extent to which these principles could be examined warrants further investigation.

In lockdown, the role of the coach in trying to encourage athletes to maintain training is important and clearly, with facilities closed, they will need to use online methods. With mood management influenced by training, identifying what strategies can be done is useful. A gap in the literature on mood regulation in general is on how coaches try to enhance the mood states of their athletes is under-examined (Friesen et al., 2013). The coach-athlete relationship seems particularly important in boxing. Interviews from nine professional boxers examining training experience found social support from other people was an important strategy (Simpson and Wrisberg, 2013). Gym partners were an integral role in this by providing advice, bonding and rivalry. Fun and humor was another dimension to this support dynamic. Research has tended to focus on self-regulation of mood rather than focusing on contextual factors and particularly how active agents such as coaches, parents or friends play a role or contribute to the mood regulation of others. An examination of an individual’s perception of self-regulation and the perception of what significant others in that context would give a broader understanding of how to help people self-regulate mood. Few studies have sought to assess coach and athlete and how they might regulate mood, something that seems particularly relevant in COVID-19, when options to regulate mood are limited.

When seen collectively, the aims of the present study are 2-fold: firstly, to investigate the mood states and explore self-regulation strategies of boxers during COVID-19, and second, to investigate how boxers and coaches adapted training methods, and sought to provide support for mood management during COVID-19.

## Materials and Methods

### Design

A mixed method design was used. The rationale for this approach was that the BRUMS is a highly validated mood measure that has been used to assess changes in mood across a number of different contexts and used successfully in the COVID-19 crisis (Lane et al., 2004; Van Wijk et al., 2013; Sties et al., 2014; Terry et al., 2020) and therefore it is possible to identify mood profiles. In contrast, the use of self-regulation strategies in the context of boxing requires open-ended questioning. Strategies to regulate emotions and adapt training in training required using an open-ended method as suitable standardized scales do not exist. A recent study in running (Stanley et al., 2012), using an open-ended questionnaire illustrated the vast array of different strategies that people use. As the present study was interested in capturing unique participants perspectives, open-ended methods are suitable. Further, via data from participants it would be possible to develop interventions grounded in participants’ experiences.

### Participants

Participants comprised 44 boxers (age: 19.4 ± 4.6 years; weight: 63.7 ± 12.9kg; experience: 4.7 ± 3.3 years; n = 33 male; n = 11 female). In terms of level of competition, 22 boxers were competing at club level, defined as participated in arranged interclub bouts. 2 boxers competed at regional level, defined as having competed in or won a regional final. 15 boxers competed at national level, defined as having participated in or won a national final. 1 boxer had competed at international level and 5 participants were professional boxers. All boxers were currently in training for a contest or competition, prior to COVID-19 lockdown. All boxers and coaches were affiliated to either England Boxing or the British Boxing Board of Control.

The second group consisted of 14 boxing coaches (*n* = 11 male; *n* = 3 female, number of years coaching 5.6 ± 5.4 years; 10 club level and 4 national level coaches). Club level defined as a coach who had coached boxers to have participated in or won a regional final. National level coaches defined as a someone who had coached previous national champions or was currently coaching a national programme.

Due to authors extended relationships with boxing clubs in the Midlands region, questionnaire invitations were sent to boxers and coaches of 6 boxing clubs within this region. Though, to supplement openness in question answers all participants names and clubs were kept anonymous. This meant individual coach-athlete dyadic relationships were not investigated.

#### Measure of Mood

The BRUMS questionnaire was used to assess feelings before and during COVID-19. The BRUMS is a 24-item scale which has been through an extensive validation process including demonstrating factorial validity, criterion validity, predictive validity, and used in many applied contexts (Terry and Lane, 2010). It has been successfully used to assess mood in combat sports including Mixed Martial Arts (Brandt et al., 2018) and Boxing (Hall and Lane, 2001). The BRUMS has 24 mood items that assess six factors: anger, confusion, depression, fatigue, tension and vigor. Example of items for each factor can be found in Table 1. For each item, participants select a number from a 5-point scale where 0 = “not at all,” 1 = “A Little,” 2 = “Moderately,” 3 = “Quite A Bit,” and “4 = Extremely.” When the mood items for each subscale are summed, a score from 0 to 16 is obtained (Terry and Lane, 2010).

**TABLE 1:** BRUMS question items for subscale scores.

Subscale	Items
Tension	Panicky, anxious, worried, nervous (items 1, 13, 14, 18)
Depression	Depressed, downhearted, unhappy, miserable (items 5, 6, 12, 16)
Anger	Annoyed, bitter, angry, bad tempered (items 7, 11, 19, 22)
Vigor	Lively, energetic, active, alert (items 2, 15, 20, 23)
Fatigue	Worn out, exhausted, sleepy, tired (items 4, 8, 10, 21)
Confusion	Confused, mixed up, muddled, uncertain (items 3, 9, 17, 24)

In the present study, a standard response timeframe of “How do you feel right now?” was used to assess mood during the COVID-19 lockdown. To assess mood before lockdown, a retrospective response time frame was used. The use of retrospective approaches has been criticized in the literature (Lane et al., 2011), but this was mainly for use in performance prediction. Retrospective measures have been criticized for being limited by the accuracy of memory, and the effect of mood at the time of recall; good accuracy has been found for using retrospective for intense moods, that is, if a person was very angry, they remember this experience and can recall it well against measures taken in real time (Winkielman et al., 1998; Terry et al., 2005). Mood measures assessed over a long period of time are more likely to assess traits or typical mood states than a response to situational factors. In the present study, mood before lockdown was not necessarily a period connected with intense mood, although, of course, there could have been situational factors that elevated mood. In addition, retrospective measures suffer lack of accuracy due to mood at the time of recall. Evidence shows that if a person is experiencing an intense, this colors their memory. And so, if you are angry at the time of completion, then recall incidents of anger much easier.

If you accept that a retrospective measure of mood is providing more of a trait measure, then this a better starting point when considering how to interpret scores (Terry et al., 2005). If the purpose of the study is to capture memories of mood, and the effect of current mood is considered, then retrospective measures become extremely useful tools as they allow capturing assessments of mood in environments where a “right now” measure is not possible. In the present study, participants completed a retrospective measure of mood before COVID started, and a measure of mood during COVID. By having the 2 completions at the same time, participants could accurately identify rate whether their mood had deteriorated. Hence, retrospective approach is not claiming to be an accurate assessment of mood before lockdown, but by taking 2 measures of mood, perceived differences could be established.

#### Procedure

Following ethical approval from the University of Wolverhampton, participant recruitment and data collection was undertaken over a 2-week period from the beginning of April 2020. Firstly, a poster was developed to advertise and encourage participation in the project. It was placed on various social media platforms and invited participants to complete an online survey via GoogleForms. When this link was clicked, both boxers and coaches were required to first read through the participant information sheet and sign the consent form. Only once this was complete could they continue to their respective sections. The use of online surveys is a credible methodology which has advantages to both researchers and participants which was recently well discussed (Braun et al., 2020). This study discusses the opportunity for a wide angle lens to capture broad perspectives on potentially sensitive topics, participants feel online surveys are unobtrusive, less burdensome and gives them a sense of control. Moreover, researchers find the data analysis less demanding than interviews and gives opportunity to explore topics where access to face-to-face research is unavailable (Braun et al., 2017)—such as during the COVID-19 pandemic.

The boxer questionnaire consisted of 3 sections including 2 BRUMS questionnaires (first BRUMS answered retrospectively before COVID-19, second in relation to emotions right now during COVID-19). Boxers were instructed to select only one number to represent the intensity of 24 emotion items before and during COVID-19. Following this, an open question section was completed regarding impacts of COVID-19, strategies used to regulate mood during COVID-19 with a focus on exploring behaviors from their coach. One question example being “What have you done to regulate your mood to deal with canceled bouts/championships and boxing sessions?” A preliminary check was undertaken to establish the subjective importance of boxing training to regulate boxer’s mood from a boxer and coaches’ perspective at the start of the open-ended question section. Asking “How would you rate the importance of boxing training toward controlling your own/your boxers’ mood and mental health?”. This was rated on a 1–5 scale where 1 = not important at all, to 5 = very important.

Coaches responded to open questions which sought to gather information on their concerns during COVID-19, methods to support boxers training, mental health and mood regulation. One example being “What are you doing to regulate your boxer’s mood during this gym closure period?”. Both boxers and coaches were instructed to answer open questionnaires as honestly and thoroughly as possible. Open questions were developed within the lens of appropriateness, clarity, unbiased and unambiguousness in question wording (Yaddanapudi and Yaddanapudi, 2019). To facilitate direct questioning toward the specific scenario of the COVID-19 pandemic, subsequent need for training modifications and the boxing participant base, it was considered relevant to include open questioning over a standardized mood regulation questionnaire. Though, questioning for both boxers and coaches were developed through considerations of interpersonal mood regulation strategies used in sport (Friesen et al., 2013) within the context of constraints presented by COVID-19.

#### Data Analysis

To examine mood state changes, scoring for the 6 subscales (Terry and Lane, 2010) was undertaken on BRUMS responses before and during COVID-19. This was undertaken only on boxers in the sample, coaches did not complete a BRUMS questionnaire. Mean ± *SD* data calculated for the preliminary check of importance of boxing training toward controlling mood. Using IBM SPSS Statistics 24 package, a repeated measures MANOVA was used to compare mood states before and during COVID-19 and to examine the extent to which depressed mood exacerbated increases in unpleasant mood. Significance level set at *p* < 0.05 for the omnibus result from the MANOVA. Effect sizes (*d*) were defined as small (0.2), medium (0.5), or large (0.8) in concordance with traditional effect size principles (Cohen, 1988).

For qualitative data, a conventional content analysis was used (Hsieh and Shannon, 2005). Initial analysis by authors sought to familiarize themselves with the data by reading both boxer and coach responses in a general manner. Generating themes individually that encapsulate underlying theory. Further to this, questionnaire responses were split into boxer only responses and coach only responses for further analysis. This was indicated in the introduction to the questionnaire which asked if the person completing was a boxer or a coach. If a response to a question had multiple strands, it was split down into appropriate codes (if longer answer) or single most appropriate (if shorter answer). Individual author boxer and coach coding were discussed then amendments to both General Dimensions and Themes undertaken. Though, initial analysis was generally agreed between authors due to majority of responses being single sentence responses rather than large bulks of text. The main discussion between authors were regarding the appropriate naming of themes to encapsulate the narrative of the study (e.g., “Adaptive Strategies” to “Promoting Positive Mood;” “Self Determination” to “Focus on Goals;” “Motivation Model of Coach-Athlete Relationship” to “Boxer-Coach Communication”). To enhance trustworthiness of the analysis, two sufficient verbatims tables of raw data examples have been provided in Tables 4, 5.

## Results

### Quantitative

A preliminary check on how important boxing was in terms of the participants’ sense of identity revealed boxing training as especially important for mental health and maintaining positive mood (boxer = 4.50 ± 0.90 and coach = 4.00 ± 078).

In terms of mood state changes from before to during COVID-19 for boxers, results indicated significantly (*p* < 0.01) higher scores for anger, confusion, depression, fatigue, tension, and lower vigor scores during COVID-19 (Table 2). However, when depressed mood was considered, it accounts for a large variance in the increase of unpleasant mood states, particularly anger, and confusion. It should be noted that depressed mood scores among boxers increased significantly during COVID-19.

**TABLE 2:** Mood states scores for boxers retrospectively before and during COVID-19.

Subsection	Pre COVID-19	During COVID-19	*F*	Cohen *d*
Anger	2.02 ± 2.16	5.29 ± 3.84	32.62	0.44
Confusion	1.43 ± 2.31	6.61 ± 4.01	57.53	0.56
Depression	1.04 ± 1.81	4.47 ± 3.90	45.11	0.49
Fatigue	2.52 ± 2.37	3.63 ± 3.24	5.93	0.11
Tension	1.27 ± 1.78	3.20 ± 3.12	23.51	0.35
Vigor	10.64 ± 3.44	7.81 ± 3.91	30.87	0.41

MANOVA results (Table 3) to examine the interaction effect for changes in mood before and during COVID-19 by depressed mood group showed no interaction effect (Wilks lambda 8, 42 = 0.94, *p* = 0.44, partial Eta^2^ = 0.06). Significant main effects were found for worsening of mood during COVID-19 (Wilks lambda 8, 42 = 0.72, *p* < 0.0001, partial Eta^2^ = 0.29) and a significant effect for depressed mood (Wilks lambda 8, 42 = 0.65, *p* < 0.0001, partial Eta^2^ = 0.35).

**TABLE 3:** Univariate results for boxers mood states by depression v no-depression during COVID-19.

	Pre-during			Depression			Pre-during × Depression		
Subsection	*F*	*P*	Partial Eta	*F*	*P*	Partial Eta	*F*	*P*	Partial Eta
Anger	3.25	0.06	0.037	38.068	0.000	0.31	1.74	0.191	0.02
Confusion	18.35	0.000	0.180	31.552	0.000	0.27	4.49	0.037	0.05
Fatigue	0.05	0.82	0.001	14.237	0.000	0.145	0.34	0.564	0.004
Tension	2.30	0.13	0.027	9.299	0.003	0.100	1.16	0.280	0.01
Vigor	5.76	0.02	0.064	2.553	0.114	0.029	0.001	0.970	0.00

### Qualitative

In line with the qualitative analysis, individual responses were split into Coach questionnaire responses (Table 4) and Boxer questionnaire responses (Table 5). As Tables 4, 5 indicate, results are presented with examples of raw data, Themes, and General Dimensions. As Table 4 indicates, coach data could be described in 3 General Dimensions of *COVID-19 Impact (Coach Perspective)*, *Coach Concerns* and *Coaches Strategies for Regulating Boxers Mood*. While boxer qualitative data could be described in 3 General Dimensions *of COVID-19 Impact (Boxers Perspective)*, *Boxer Perceived Coach Supportive Behaviors and Regulatory Strategies used by Boxers.*

**TABLE 4:** Analysis of Coach responses on the impact of COVID-19 on training and competition, coach concerns and strategies to regulate boxers mood.

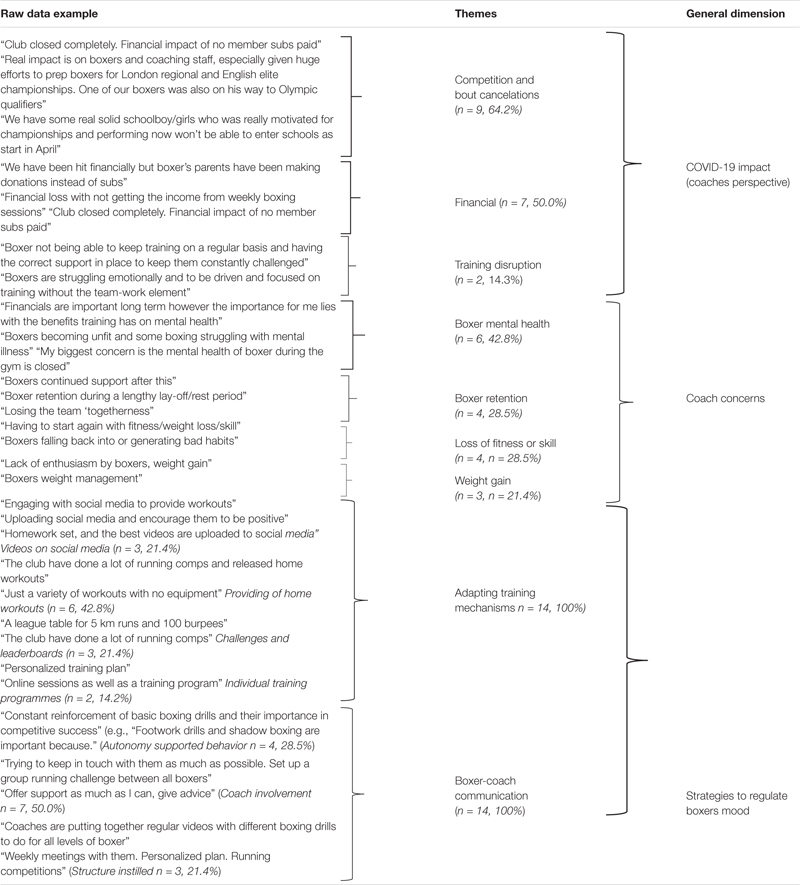

**TABLE 5:** Analysis of boxer responses on the impact of COVID-19 on training and competition, boxer perceived coach supportive behaviors and strategies for regulating their own during COVID-19.

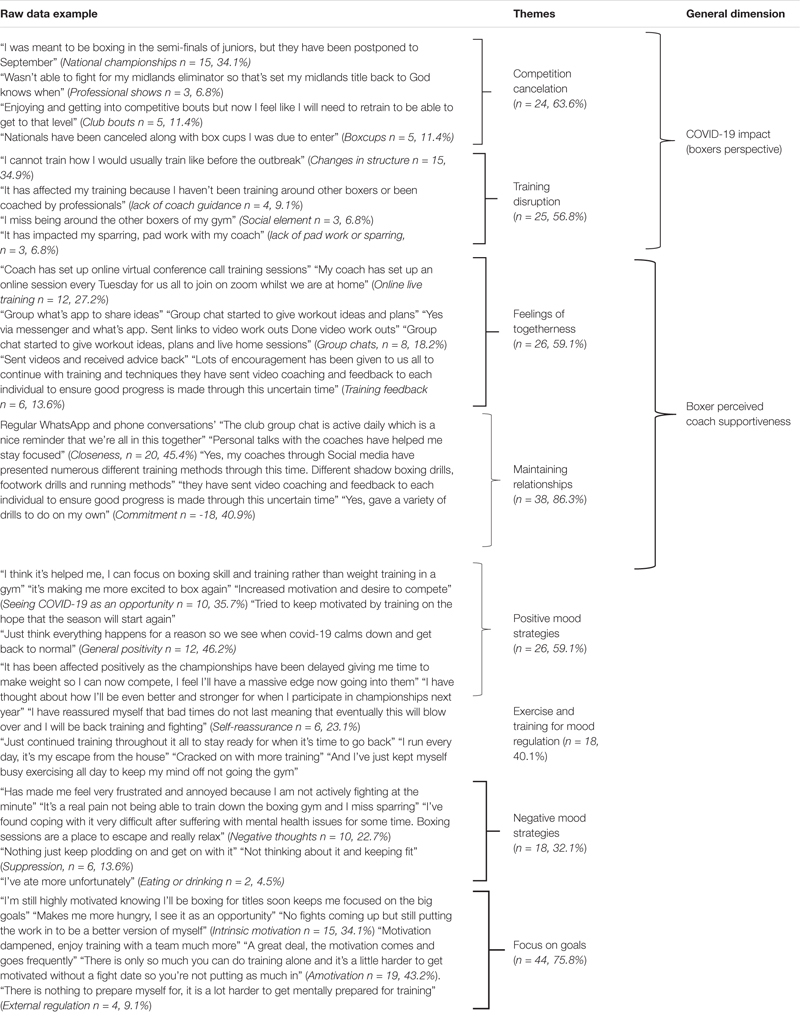

As Table 4 indicates, the first General Dimension *COVID-19 impact (Coach Perspective)* encompassed 3 themes labeled *Competition and Bout Cancelation, Financial Impact and Training Disruption. Coach Concerns* consisted of themes relating to *Boxer Mental Health*, *Boxer Retention* post COVID-19, *Loss of Fitness or Skill* and *Weight Gain*. Whereas *Strategies to regulate boxer’s mood* consisted of *Adapting Training Mechanisms* and maintaining *Boxer-Coach Communication*.

As Table 5 indicates, *COVID-19 (Boxer Perspective)* encompassed 2 themes relating to *Competition Cancelation* and *Training Disruption*. *Boxer Perceived Coach Supportiveness* could be described into 2 themes of *Feelings of Togetherness* and *Maintaining Relationships*. Whereas *Regulatory Strategies used by Boxer*s had 3 themes of *Positive Mood Strategies*, *Negative Mood Strategies* and *Focus on Goals*.

#### Coaches Strategies for Regulating Boxer’s Mood

Qualitative results for data collected from coaches indicated that at the outset of the pandemic, coaches began to work online to help guide training (see Table 4). The theme *Boxer-Coach Communication* was split to further themes including *Autonomy Supportive Behaviors*, *Coaches Involvement* and *Instilling Structure*. Setting of group challenges to promote internal competition, online check ins (e.g., via zoom) and weekly support in training group chats (e.g., WhatsApp) were key strategies coaches used to regulate boxers.

#### Boxer Perceived Coach Supportiveness

Boxer perceived Coach Supportiveness comprised of statements of boxer feelings of *Closeness* with coaches through regular contact. This closeness was personified when one boxer stated, “without my coaches I’d be totally lost.” In contrast, some boxers reported that coaches offered limited/no supportive guidance or advice during COVID-19 (Table 5). Whereas *Commitment* shown by coaches toward adapting training structure and guiding toward future success was recognized by boxers. With one example being “they (coaches) have sent video coaching and feedback to each individual to ensure good progress is made through this uncertain time”.

#### Regulatory Strategies Used by Boxers

##### Positive mood strategies

*Positive Mood Strategies* comprised 3 themes. Firstly, *seeing COVID-19 as an opportunity*. This consisted of responses relating to extended time to make weight, train and work on weaknesses, for example, “I have told myself this is just a way to give me more time to prepare.” Secondly, *General Positivity*, for example, “I feel as though my boxing training is going well considering the situation.” Thirdly, *Self-Reassurance*, whereby boxers reassured themselves with affirmations about the future (e.g., “I have reassured myself that bad times don’t last”). Reassurance was extended toward future competitions, for example, “I have thought about how I’ll be even better and stronger for when I participate in championships next year.”

*Exercising and Training for Mood Enhancement.* For many boxers, the training week was a key strategy to regulate mood within itself for their physical and mental wellbeing. With a boxer reporting “I run every day, it’s my escape from the house.” While others reported they stayed in a similar routine to their usual training days and times (Table 5).

##### Negative mood strategies

*Negative Mood Strategies* widely consisted of *Negative thoughts* toward struggles with mental health and frustration with training modification. This was further unpacked by boxers who indicated it was caused by limitations in space, necessary equipment to train optimally, lack of face-to-face professional guidance and limited social interactions with teammates (Table 5). These circumstances were acknowledged by one boxing coach who reported “Boxers are struggling emotionally and to be driven and focused on training without the teamwork element.”

*Suppression* related responses consisted of not acknowledging current feelings toward COVID-19 and engaging with a narrative that avoided acknowledging the situation, for example, “Nothing just keep plodding on and get on with it.” A small sample of boxers described attempting to regulate mood through *Eating/Drinking* during COVID-19.

##### Focus on goals

During COVID-19 the focus on goals set for the season lacked focus and not surprisingly, with no identified date for when sport would return, many expressed a lack of motivation to maintain training (see Table 5), for example, “With no fight date, so find yourself going into a tick over mode.” Although, trying to focus on potential future success was a strategy used by some boxers to ensure they continued training during the pandemic. One boxer responded by saying; “I’m still highly motivated knowing I’ll be boxing for titles soon keeps me focused on the big goals.”

## Discussion

The present study investigated mood states and self-regulation strategies of boxers and mood regulation strategies used by coaches at the start of the COVID-19 outbreak and first month of UK lockdown. The study makes a valuable contribution to the literature as it details mood states and regulation strategies used by boxers and coaches during the first COVID lockdown in the UK, which itself represents a unique set of circumstances. The authors argue that an effective method to examine this issue was via a combined use of mixed method using self-report data via responses to a standardized scale along with using Open ended questions to capture mood regulation strategies (Friesen et al., 2013). Results indicated that the restrictions and changes that accompanied COVID-19 were associated with a substantial increase in unpleasant emotions. Findings are consistent with recent research that has used the BRUMS during COVID-19 (Richardson et al., 2020; Terry et al., 2020). Using recent mood profiles, mood during lockdown resembled an inverse iceberg profile. In the present study, it unpacked unpleasant mood by the influence of depression using Lane and Terry’s (2000) model. Our combined findings show mood disturbance occurred. Results show boxers and coaches were actively seeking to manage mood. A key aspect of our study is also to focus on boxing, a sport that is under-reported in the literature. Arguably, the mood states of boxers in training and how they regulate mood represents a novel line of research.

Using Lane and Terry’s (2000) conceptual model to investigate mood states by the presence and absence of depression, where the notion is that depressed mood associates with an overall negative mood state, the present study found 70.4% of boxers reported depressive symptoms during COVID-19, a figure that is substantially greater than the mode reported in a review of the model (Lane and Terry, 2016). In a study of 44,742 participants, depressed symptoms were present in only 25% of participants (Lane et al., 2017) suggesting the effects of COVID-19 were substantial, as expected. The results demonstrate the expected effects of COVID-19 where mental health concerns are predicted (Winkielman et al., 1998). Our findings are consistent with previous tests of Lane and Terry’s model which show symptoms of depressed mood corresponds with a general negative mood profile (Lane et al., 2017). Recent work that has developed new mood profiles could illustrates new approaches to interpreting mood. To date, researchers have not investigated cluster profiles by depression group (Parsons-Smith et al., 2017; Quartiroli et al., 2018; Han et al., 2020). The submerged and surface mood profiles (Parsons-Smith et al., 2017; Quartiroli et al., 2018; Han et al., 2020) both report low depression scores and we suggest that there is a need to explore mood profiles when participants report zero for no-depression, especially as Lane and Terry (2000) suggested and previous research has supported the notion that anger and tension can have motivational effects.

As these results were expected, qualitative data provides a useful insight into why mood states worsened, what strategies boxers sought to manage their own mood (Table 4) and how coaches supported the mood states of their athletes (Table 5). The use of mixed methods for obtaining mood scores from a frequently used scale (Terry and Lane, 2010) and open-ended questions facilitate assessing the magnitude of mood state changes and then identifying strategies used to see how coach and athlete sought to manage mood. It is important to acknowledge that significant changes to boxers’ routines occurred at the outset of the COVID-19 lockdown. The effects were broad and substantial including periods of inactivity, largely connected to the initial uncertainty from the unexpected lockdown (e.g., “Elite championships canceled ruined my season” “as there is nothing to prepare myself for, it is a lot harder to get mentally prepared for training”).

The sudden withdrawal of the place where boxers train and the people they train with coupled with mass media messaging to stay at home (Flanagan et al., 2020). Consequently, there were substantial changes in the social networks of boxers and so coach support was adapted, with fewer and different social interactions, with clear physical distancing from the athletic community (e.g., “Motivation dampened, enjoy training with a team much more”). An intriguing aspect of findings from the present study is the extent to which they could be specific to boxing and might not apply to other sports than possibly other combat sports. Boxing is a sport that combines the intensity of 1-2-1 competition alongside a need to maintain tight control over body weight, a feature shared by combat sports. It is possible that training helps maintain a positive body image, which has been found to correlate with self-esteem (Franchini et al., 2012) and mood (Lane, 2006). Qualitative data (see Table 5) shows boxers held serious concerns on how they could maintain fitness without access to specific equipment (e.g., “My training has been effected very much. Can’t train much because I don’t have the correct equipment at home” “Could not train at a higher standard as usual due to not having necessary boxing equipment also cannot go outside as frequently”). This view is not entirely unsurprising as boxers would have considerable routines connected with training and none of these became possible to use during lockdown. Further, coaches expressed concerns that detraining would occur and sedentary behavior was likely during the initial lockdown period. A consequence of de-training is that weight could increase with muscle mass reducing (Paoli and Musumeci, 2020). Potentially contributing to boxers’ intense negative mood via reduced body image.

Qualitative data suggests that intensely unpleasant moods during COVID-19 (Table 2) were attributable toward changes in training structure, daily routine, social isolation, and the competition landscape being extremely uncertain. Further, limited boxing specific training equipment and space was a recurring theme (e.g., “Can’t train much because I don’t have the correct equipment at home” “I have little to no boxing equipment”). Frustration stemming from difficulties in training clearly associated with unpleasant mood and the authors suggest that this stress response was unhelpful. Stress can impede one’s effort to maintain physical activity and can have a biopsychosocial influence on fatigue (Stults-Kolehmainen and Sinha, 2013). In the presence study, participants reported significantly higher fatigue (Table 3) when it coupled with depressed mood, it suggests that the negative self-schema associated with depressed mood influenced perceptions of available energy. De-coupling fatigue from depression is important theoretically and practically and one reason the authors do not argue for calculating a total mood disturbance score, which is derived from taking vigor from the sub of anger, confusion, depression, fatigue, and tension scores. High fatigue could be explained by intense training, and so a boxer who sought to regulate mood by training harder, might experience intense fatigue but not depression. By contrast, a boxer who feels depressed, disengages with training, and reports a general sense of lethargy and report a high score of fatigue. By investigating fatigue separately from depression, this facilitates examination of how mood states could possibly interact (Lane and Terry, 2000).

It should be noted that vigor reduced during COVID-19 in the present study (Table 2) and this occurred independently of depressed mood. It could be that lockdown measures, which restricted the availability of several mood regulation activities coupled with the curtailing of ambitions to achieve seasons goals could have been relevant. It is important to remember some boxers were concluding training for the upcoming National Amateur Boxing Association Championships – a key tournament on the national circuit and as results indicate, boxing was an important aspect of personal self-worth. As qualitative data indicates, boxers held goals in respect of the forthcoming national championships, and therefore, the timing of the lockdown was particularly poor for boxers (e.g., “It has been affected because I can’t participate this year in nationals and I was mentally prepared for them”). Whilst a lockdown is always likely to be difficult, if one happened at the end of the boxing season, around the month of May, then the effects could have been less severe. However, it is worth noting that mental health in athletes has been reported to be fragile and even in usual training circumstances, 34% of elite athletes had depression and anxiety symptoms (Gouttebarge et al., 2019).

It is noteworthy some boxers responded more positively to the restrictions, adapting training and creating a more positive attitude (Table 5). For example, quotations such as “I think it’s helped me, I can focus on boxing skill and training rather than weight training in a gym” illustrate a shift in goal and moving focus to goals that are attainable in the current context, that is without equipment. Moreover, some boxers were not in optimal condition prior to lockdown, so the outlook was positive. This is encapsulated by one response “It has been delayed giving me time to make weight so I can now compete, I feel I’ll have a massive edge now going into them (national championships).” At the time, this boxer held positive beliefs that the championships would occur later in the year, and evidence shows that expecting positive future events is an effective mood regulating strategy (Restubog et al., 2020).

The present study investigated how coaches responded to managing their boxers training environment and mood states. Results show that coaches went to great lengths to modify training and maintain relationships. Such a finding is consistent with the notion that boxing coaches typically have a close personal relationship with their boxers (Simpson and Wrisberg, 2013). A great deal of boxing coaching has 1-2-1 interactions such as pad work. Further, the coach provides a critical role preparing a boxer for competition when they experience intense pre-competition emotions and continues to provide meaningful support when providing feedback to boxers between rounds in competition (Lane, 2006; Simpson and Wrisberg, 2013). Furthermore, intense bout and corner experiences will enhance the closeness, something that is conceivably a key support mechanism for boxer’s mental health in their usual training circumstances. This could explain the high subjective importance of participation in boxing training for managing boxer’s mood and mental health.

Whilst a positive relationship between coach and athlete can have many benefits (Davis et al., 2019; Trigueros et al., 2019) the speed of changes during COVID-19 represented a massive and unexpected disruption. What has emerged during the COVID-19 crisis is the development of online resources to support coach-boxer relationships (Tjonndal, 2020). However, these were not in place at the start with face-to-face being the common method of interaction. Therefore, this perceived closeness was still unable to prevent the negative mental impacts of COVID-19 which was likely to be outweighed by factors including altered training regime, training environment, sleep patterns, social isolation, poor mechanisms of support and general uncertainty (Brooks et al., 2020; Terry et al., 2020).

It should be noted that the role of supporting boxer’s mood states is one that coaches typically do specifically in relation to boxing. Results of the present study show that performance management strategies play an important role in mood management and so when exploring how boxers regulate mood, it is important to assess perceptions of training status. The present study offers this data from boxer and coaches perspective, although an acknowledged limitation is that coach-athletes data is not dyadic. Boxers results have relevance to boxing coaches in general as there is no reason to suspect that such findings would not be representative. In terms of the challenge to boxers and coaches, at the start of the lockdown, they were asked to work online, and so in an unfamiliar environment. The data captured in this study represents dynamic changes as coaches and boxers sought to create new ways of working but maintain COVID-19 specific restrictions. It should not be surprising that coaches did not have a toolkit of strategies to call upon for use. However, qualitative data suggests that they began to explore ways to help motivate their boxers that would have been modified via practice (Table 4).

Both qualitative and quantitative results of the study show how challenging boxers found regulating mood states during COVID-19. Despite coaches clearly evidencing their support and understanding toward the importance of training for boxer’s mental health, 32.1% of boxers adopted negative mood strategies during COVID-19 (Table 5). It is possible that these responses could have been driven by the mental health implications of home confinement and unrelenting flow of virus media coverage, often fueled by information and misinformation from social media networks, with panic derived from social media believed to travel faster than the outbreak itself (Depoux et al., 2020). Furthermore, 15.9% of boxers reported receiving no coach support or guidance over COVID-19, with the absence of this support mechanism potentially leveraging use of negative mood strategies.

The present study had limitations that should be considered. The participant base was solely from Midlands based boxing clubs. Exploring other regions of the UK could have enhanced generalizability of the findings. However, the authors had no reason to suspect geographical differences would have changed the results. This might change if the crisis was going into winter where weather might restrict opportunities to exercise outdoors. Second, there were more males than females, however, findings reflect the proportion of males and females in boxing, and it should be noted that there are very few female coaches in boxing. Third, participants completed their initial BRUMS questionnaire retrospectively to describe their mood states prior to COVID-19. Potential factors that influence recall include tiredness, personality traits and mood at time of reflection influences emotion recalling (Winkielman et al., 1998; Terry et al., 2005). It has also been indicated measures that summarize mood over time can be heavily influenced by the mood at the time of recall (Lane et al., 2011). They also indicated that retrospective measures are better understood as the perception of mood over that period rather than an accurate record of mood. Finally, the structure of the questionnaire meant all participants names and clubs were kept anonymous meaning individual coach-athlete dyadic relationships were not investigated. Though, this was justified by the authors to maintain openness and honesty from boxers and coaches on what could be conceived as a sensitive topic.

### Implications for Practice

Findings from the present study show the importance of identifying mood states and how people regulate their mood. The first implication for practice is to encourage regular monitoring of mood which is something that is possible via online platforms such as In the Mood^1^ which offers immediate assessment and has self-help pointers for mood regulation. Mood identification and monitoring can have meaningful effects on performance as highlighted in a study which monitored emotional state changes during Karate competition (Friesen et al., 2017). This study suggested that coaches’ ability to accurately detect the emotions of competitors help them perform better. In the present study, and in respect to contexts where negative mood might be predicted, coaches could play a meaningful role in early detection of negative mood as therefore could help prevent poor mental health developing. The present study suggests that daily mood monitoring would be useful beyond the COVID crisis.

Second, findings suggest that boxing coach education should focus on helping coaches maintain good mental health. In the lockdown, positive steps have been made to promote coach education online. Knowledge that progress in training affects mood is relevant and coaches should seek to gain an understanding how well they can identify the mood states of their athletes (Friesen et al., 2017). It is suggested that such skills would be useful for boxing coaches outside of COVID-19 due to the influence of mood states on performance.

A third suggestion is to continue to develop online communities that encourage a community of practice. A combined package of a website and use of social media to hold discussion on a range of issues from sport psychology to nutrition issues and has allowed coaches to interact.^2^^3^ The lockdown accelerated the development of the online community and given restrictions, people had the opportunity and availability to attend. Further, former professional boxers^4^ have begun encouraging boxing to be mindful of mental health and via sharing such experiences, could help boxers use self-regulation strategies more purposefully. The authors argue that such development offers a good starting point for a training programme that could be accessed online, which if there are to be further lockdowns boxing coaches would have peer-group expertise to help support their boxers.

A fourth suggestion to help mood management is to encourage boxers to use self-regulation skills. The connection between using psychological skills and mood management represents a good starting point as there is a wealth of information available offering basic guidance on using psychological skills (Karageorghis and Terry, 2010) and previous research has shown that people can learn these online (Lane et al., 2016c). We suggest, therefore, that one option available to help mental health of boxers could be to promote the use of mental skills training, which as the results of the present show, many boxers use a version of psychological skills to help mood management (Lane et al., 2012, 2016a; Stanley et al., 2012). Recent research has suggested that athletes and coaches should learn to use psychological skills, suggesting that such knowledge is analogous to knowing about good training habits, nutritional practice, and knowing these skills is helpful. When an athlete or coach needs to use the skills, they are at a more advanced stage to solve a particular issue so they are are less likely to be inhibited by learning issues. As suggested recently, and evidenced, it is possible to learn psychological skills in a brief format and investigate their effectiveness with a view to develop further learning and usage (Lane et al., 2016a, b, c). Psychological skills such as imagery, relaxation, self-talk and attentional control are worth learning as they can be applied to many different contexts (Lane et al., 2012). Mental skills offer an opportunity to work through a demanding situation and so enable the person to be better prepared when or if they meet it for real. The authors suggest that mental training should be taught as routine practice, just as an athlete should have good knowledge of factors such as training theory and nutrition so that they can make informed decisions on how to engage in self-regulation. The present study indicated how concerns for diet, reductions in fitness and changes in training contributed to negative mood. This point seems particularly apt as the implications of COVID-19 are that there is a second wave which has just started at the time of submission (October 2020). Helping boxers and coaches develop mental strength seems particularly important as they are predominantly from socially deprived backgrounds, and this makes them more vulnerable to mental health problems (Reiss et al., 2019).

The strategies learned from a coach’s psychological skills programme could be structured into current training by coaches via online mechanisms, which have continued to grow throughout COVID-19. With the amateur boxing competition landscape unknown, developing psychological skills and improving mental health for future seasons, could be fruitful endeavors. Further, and when COVID-19 restrictions pass, integrated online mechanisms could make boxers, parents and coaches more reachable. This would create a sense of community and belonging that should outlast the current crisis and help protect people during the second wave if a boxer/coach must self-isolate due to COVID-19. During these extremely uncertain times and the mental impacts of COVID-19 likely to outlast the physical illness (Paoli and Musumeci, 2020), these practices could be more crucial than ever.

## Conclusion

To conclude, results suggest COVID-19 had a significant negative impact on boxers’ mood state, a profile that was significantly more unpleasantly if participants reported symptoms of depression. Qualitative data indicated boxers were affected by changes in training, a finding that suggests how closely boxing training plays in mood regulation. Qualitative data from coaches showed a similar trend, recognizing that training is likely to affect mood. It is suggested that future research should help boxers self-regulate mood and coach identify mood state changes in boxers, and moves to provide online support for boxers and coaches that emerged are positive developments that should continue post pandemic.

## Data Availability Statement

The original contributions presented in the study are included in the article/supplementary material, further inquiries can be directed to the corresponding author/s.

## Ethics Statement

The studies involving human participants were reviewed and approved by the University of Wolverhampton. The patients/participants provided their written informed consent to participate in this study.

## Author Contributions

RR responsible for data collection process (inputting mood scores, depression scores criteria using Lane and Terry Model, inputting qual data), completion of qualitative data analysis, parts of the SPSS data analysis (mood scores pre and during COVID), manuscript write up of results and completion of tables. Contributions to initial write up of introduction, method and discussion. AL responsible for quantitative data analysis (MANOVA), refining and amending introduction, method, results and discussion write up sections. Both authors contributed to the article and approved the submitted version.

## Conflict of Interest

The authors declare that the research was conducted in the absence of any commercial or financial relationships that could be construed as a potential conflict of interest.

## Footnotes

1 https://www.moodprofiling.com/testRP-S.php

2 https://twitter.com/boxgathering

3 https://www.youtube.com/channel/UC_Hz9bo30cUIW5NHBaViozg

4 https://billyschwer.com/
